# Beyond a mirror-image procedure: a case report of leadless pacemaker implantation in dextrocardia with situs inversus

**DOI:** 10.1093/ehjcr/ytaf674

**Published:** 2025-12-23

**Authors:** Kohei Iwasa, Masato Okada, Yuki Masuda, Yasushi Koyama, Nobuaki Tanaka

**Affiliations:** Cardiovascular Center, Sakurabashi Watanabe Advanced Healthcare Hospital, 4-3-51 Nakanoshima, Kita-ku, Osaka 530-0005, Japan; Cardiovascular Center, Sakurabashi Watanabe Advanced Healthcare Hospital, 4-3-51 Nakanoshima, Kita-ku, Osaka 530-0005, Japan; Cardiovascular Center, Sakurabashi Watanabe Advanced Healthcare Hospital, 4-3-51 Nakanoshima, Kita-ku, Osaka 530-0005, Japan; Cardiovascular Center, Sakurabashi Watanabe Advanced Healthcare Hospital, 4-3-51 Nakanoshima, Kita-ku, Osaka 530-0005, Japan; Cardiovascular Center, Sakurabashi Watanabe Advanced Healthcare Hospital, 4-3-51 Nakanoshima, Kita-ku, Osaka 530-0005, Japan

**Keywords:** Leadless pacemaker, Sick sinus syndrome, Dextrocardia with situs inversus, Case report

## Abstract

**Background:**

Dextrocardia with situs inversus (DC + SI) is a rare congenital anomaly characterized by mirror-image positioning of the heart and thoracoabdominal organs. Leadless pacemaker (LP) implantation in patients with DC + SI presents unique anatomical and technical challenges, and clinical experience remains limited.

**Case summary:**

A 76-year-old woman with DC + SI presented with tachycardia–bradycardia syndrome. Given her symptomatic bradycardia, we undertook LP implantation (Micra, Medtronic, Minneapolis, MN, USA). Pre-procedural computed tomography demonstrated a mirror-image arrangement of the thoracoabdominal organs including the heart in the right hemithorax, establishing the diagnosis of DC + SI. During implantation, conventional mirror-image catheter manipulation—counterclockwise rotation mirroring clockwise rotation in normal anatomy—caused the catheter tip to deflect towards the right ventricular free wall, due to the pre-shaped curvature of the Micra delivery catheter. Additional counterclockwise torque was required to redirect the tip towards the septum for successful deployment. The procedure was completed without complications, and the patient remained asymptomatic with stable device parameters at the 1-year post-implantation.

**Discussion:**

This case highlights how the pre-shaped curvature of the Micra delivery catheter introduces procedural complexity beyond a simple mirror-image manipulation. Additional counterclockwise torque is often required to achieve septal positioning. Comprehensive pre-procedural imaging and an understanding of the device-specific characteristics are essential for successful LP implantation in these anatomically reversed cases.

Learning points

- The three-dimensional curvature of the Micra delivery catheter adds procedural complexity beyond simple mirror-image manipulation in dextrocardia.
- Additional counterclockwise torque is essential to overcome the pre-shaped curvature and ensure correct septal deployment in mirror-image anatomy.
- Awareness of the tip and shaft behaviour during counterclockwise torque application is crucial for safe and accurate implantation in anatomically reversed hearts.

## Introduction

Dextrocardia with situs inversus (DC + SI) is a rare congenital anomaly in which the heart and thoracoabdominal organs are positioned in a mirror-image configuration.^1–3^ Due to its rarity, clinical experience with leadless pacemaker (LP) implantation in patients with DC + SI is limited, and the associated technical challenges remain poorly defined.^4–6^ Here, we present a case of successful Micra (Medtronic, Minneapolis, MN, USA) LP implantation in a patient with DC + SI, highlighting key procedural considerations and device-specific handling strategies.

## Summary figure

**Figure:**
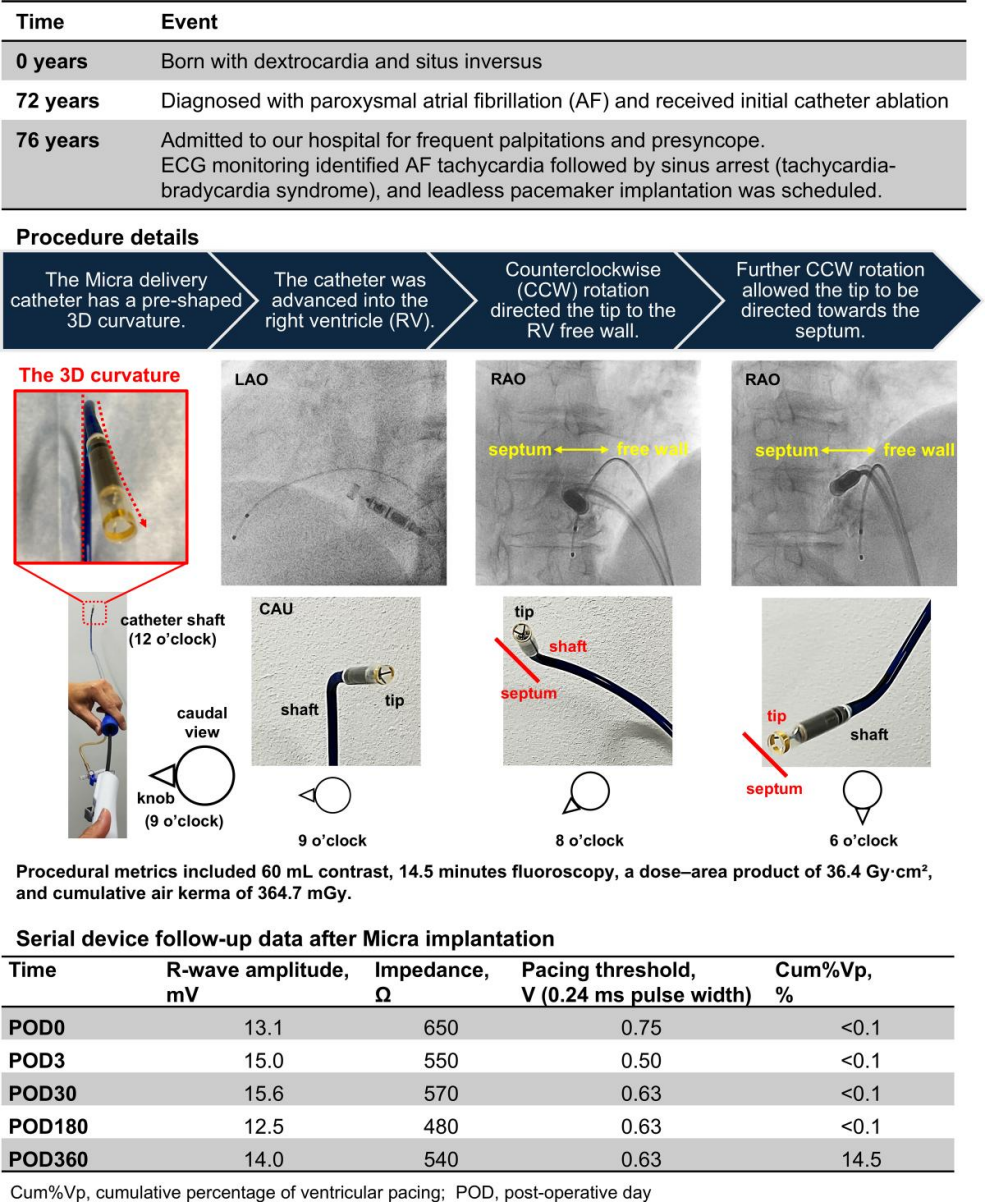


## Case presentation

**Figure 1:**
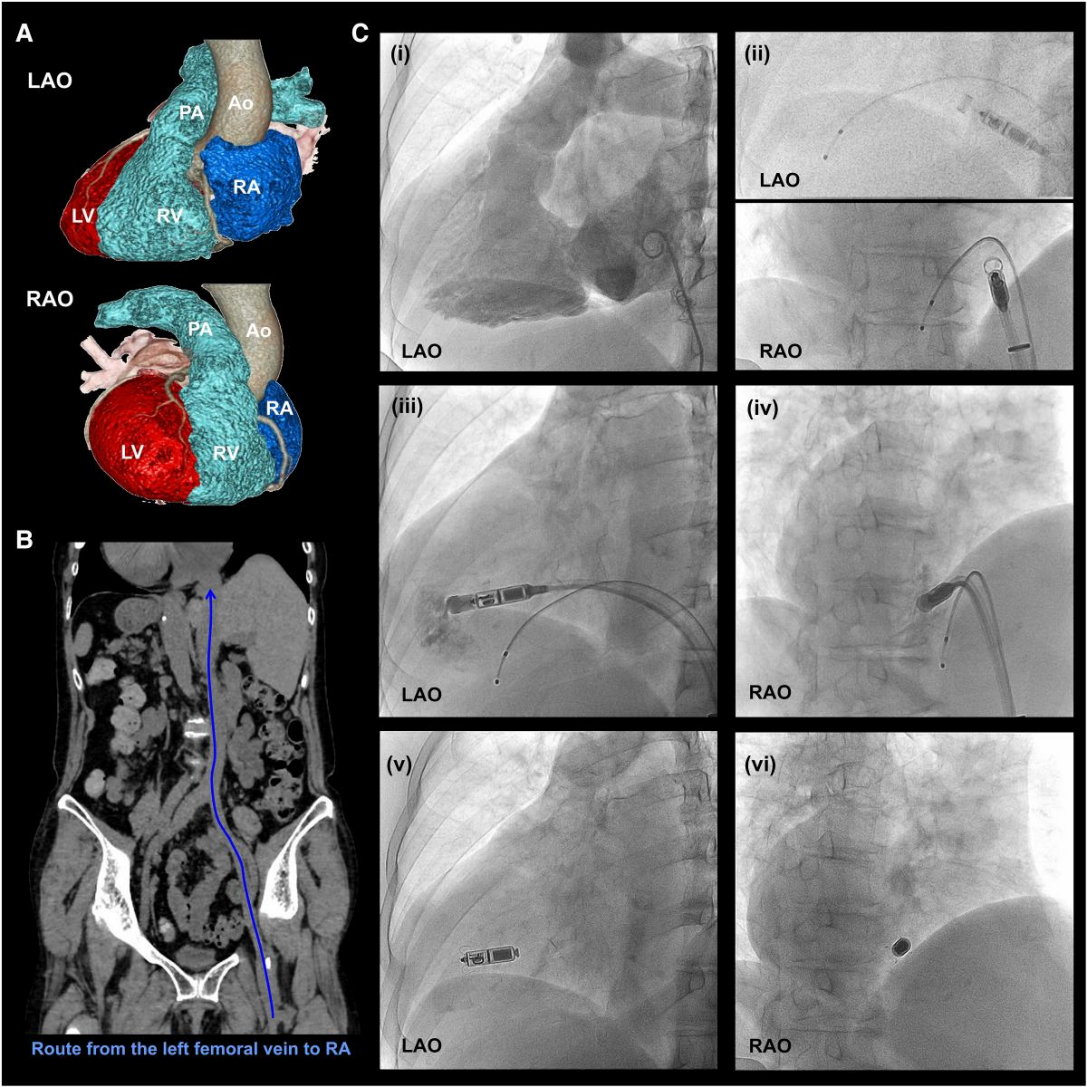
Pre-procedural imaging and fluoroscopic views during Micra implantation. (*A*) Three-dimensional reconstruction image of the heart created using a multidetector computed tomography. (*B*) Coronal curved computed tomography planar image showing the venous pathways from the groin to the right atrium, revealing a straight route from left femoral vein to the right atrium (blue arrow). (*C*) Fluoroscopic images during Micra implantation. (i) Right ventriculography. (ii) Advancement of the Micra delivery catheter into the right ventricle using a temporary pacing catheter for guidance, confirmed in right anterior oblique and left anterior oblique views. (iii and iv) Successful catheter positioning at the lower septum using additional counterclockwise torque, confirmed with contrast. (v and vi) Device deployment in right anterior oblique and left anterior oblique views. Ao, aorta; CT, computed tomography; FV, femoral vein; IVC, inferior vena cava; LAO, left anterior oblique; LV, left ventricle; PA, pulmonary artery; RA, right atrium; RAO, right anterior oblique; RV, right ventricle.

A 76-year-old woman previously diagnosed with DC was admitted to our hospital for frequent palpitations and presyncope. She had previously undergone three atrial fibrillation (AF) ablations, but the electrocardiogram monitoring revealed AF tachycardia followed by sinus arrest (i.e. tachycardia–bradycardia syndrome), which corresponded to her symptoms. Bradycardia was considered irreversible due to the absence of electrolyte imbalance or antiarrhythmic medication use. Transthoracic echocardiography revealed normal left ventricular function without significant valvular dysfunction or other cardiac anomalies. Pre-procedural computed tomography (CT) demonstrated a mirror-image arrangement of the abdominal organs and right-sided heart, thereby confirming the diagnosis of DC + SI. Three-dimensional CT reconstruction of the heart, aorta, pulmonary artery, and inferior vena cava (IVC) further delineated the reversed anatomy (*Figure 1A*). The IVC was located to the left side of the spine, rendering the left femoral vein (FV) approach anatomically equivalent to conventional right FV access (*Figure 1B*).

Micra LP implantation was performed to treat symptomatic bradycardia. The procedure was carried out under standard non-flipped fluoroscopic orientations. Following bilateral FV access, right ventriculography was conducted using a pigtail catheter (*Figure 1C* (i)). Subsequently, a 5-Fr temporary transvenous pacemaker was placed at the right ventricular (RV) apex via the right FV as a guide for the Micra delivery catheter. In contrast, a 27-Fr delivery sheath (Medtronic, Minneapolis, MN, USA) was inserted via the left FV under fluoroscopic guidance. Afterward, the Micra delivery catheter was advanced into the right atrium (RA) and rotated counterclockwise under the 30° right anterior oblique view, ensuring that the catheter shaft was parallel to the temporary catheter (*Figure 1C* (ii)). The catheter tip was then carefully advanced across the tricuspid valve into the RV.

Initially, the tip of the delivery catheter repeatedly deflected towards the RV free wall or slipped inferiorly from the septum towards the hinge or the inferior wall (*Video 1*). However, by slightly retracting the delivery system and applying additional counterclockwise torque, the catheter tip was successfully positioned at the lower septum near the apex. Contrast-enhanced fluoroscopy confirmed a safe margin from the hinge (*Figure 1C* (iii and iv)), and stable fixation was achieved at this site on the first deployment (*Figure 1C* (v and vi)). After confirming the fixation stability by the pull-and-hold test, the tether was cut and the device released. Acute parameters were as follows: sensed R-wave amplitude, 13.1 mV; impedance, 650 Ω; and pacing threshold, 0.75 V at 0.24 ms. The device was programmed to VVI 40 ppm. Procedural metrics included 60 mL of contrast, 14.5 min of fluoroscopy, a dose–area product of 36.4 Gy cm², and cumulative air kerma of 364.7 mGy. The patient was discharged without complications and remained free from presyncope with stable pacemaker parameters at the 1-year follow-up (*Summary figure*).

## Discussion

We successfully implanted Micra LP in a patient with DC + SI. Initially, mirror-image manipulations caused the catheter tip to deviate towards the RV free wall or slip inferiorly from the septum. However, by slightly retracting the catheter and applying additional counterclockwise torque, the tip was directed towards the septum, allowing stable deployment near the lower septum. This technical challenge can be attributed to the inherent design of the Micra delivery catheter.

**Figure 2:**
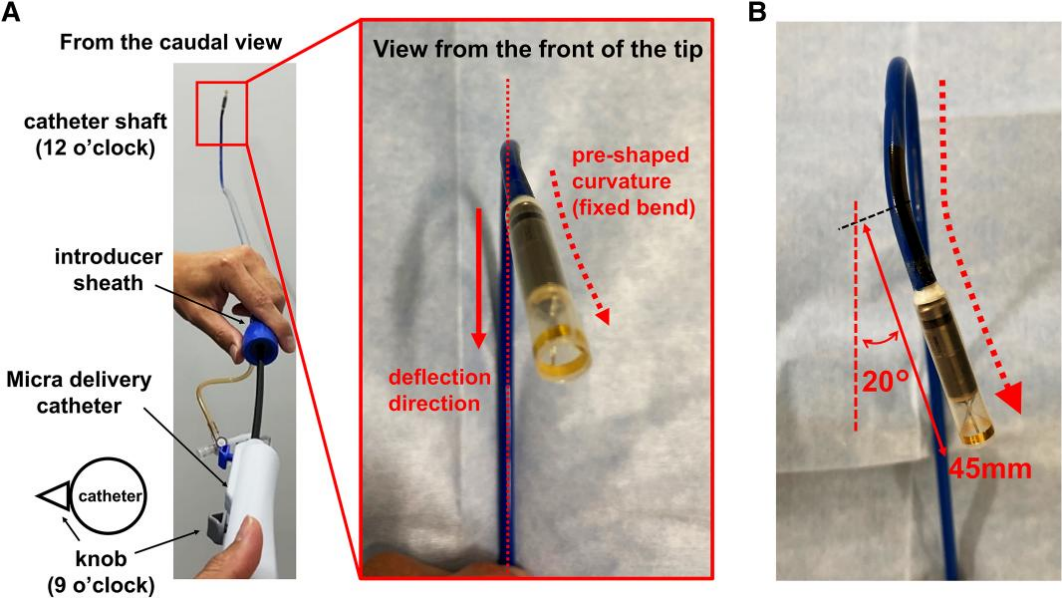
Three-dimensional structure of the Micra delivery catheter. (*A*) Left panel: Illustration of the introducer sheath and Micra delivery catheter with the deflection knob positioned at 9 o’clock. Right panel: Caudal view showing the angular discrepancy between the catheter shaft (solid red arrow) and pre-shaped distal tip (dotted red arrow), creating a three-dimensional curvature. (*B*) Schematic illustration of the angular deviation between the tip and shaft when the deflection knob is pulled. The distance from the tip to the bending point is 45 mm, forming an angle of 10–20°, depending on the degree of deflection. These measurements were manually assessed by the authors.

As illustrated in *Figure 2*, the Micra delivery catheter has a compound, three-dimensional curvature created by two elements: deflection of the catheter shaft and a fixed pre-shaped bend at the distal tip. When the deflection knob is positioned at 9 o’clock, the catheter shaft aligns vertically (12 o’clock position from the caudal view) (*Figure 2A*, left panel). However, from a frontal perspective, the tip curves rightward relative to the shaft, creating a three-dimensional curvature (*Figure 2A*, right panel). Further deflection accentuates this angular deviation, which must be carefully considered when navigating the catheter towards the septum in mirrored anatomy (*Figure 2B* and *Video 2*).

**Figure 3:**
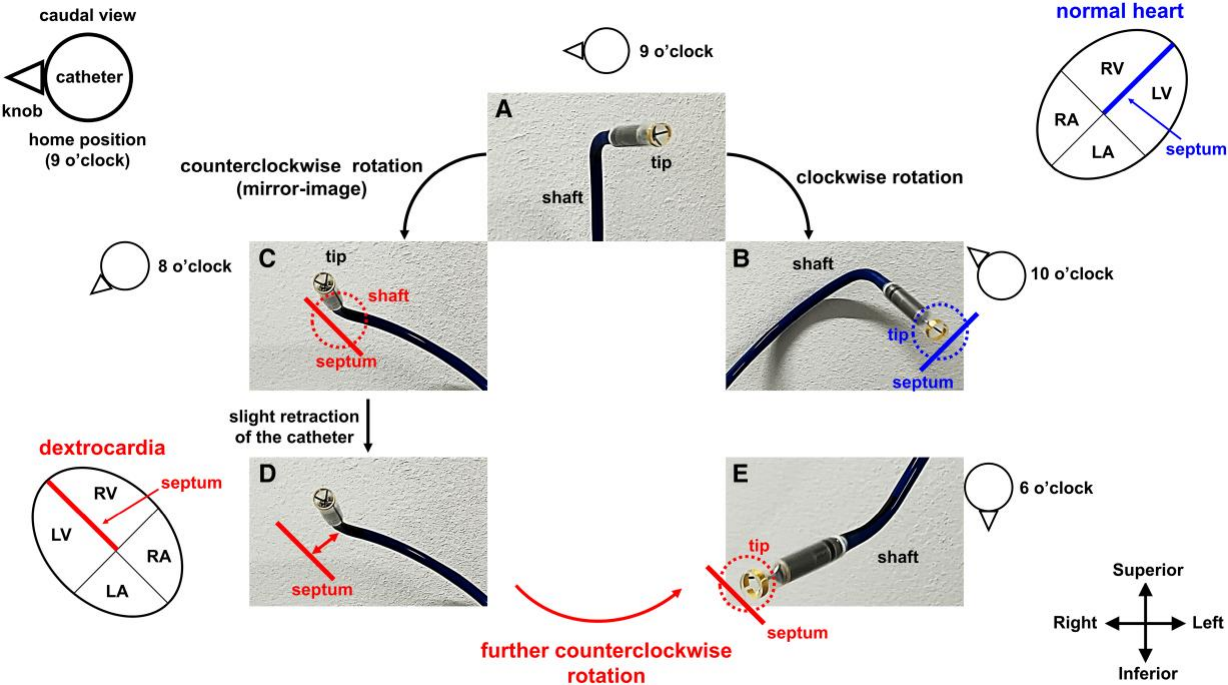
Catheter rotation and tip orientation for septal deployment in normal versus mirror-image anatomy. Schematic illustrations of device-specific handling strategies viewed from the caudal perspective. (*A*) In the neutral or ‘home’ position (deflection knob at 9 o’clock), the catheter shaft points superiorly (12 o’clock), while the pre-shaped tip deflects rightward (approximately 3 o’clock). (*B*) In normal anatomy, clockwise rotation from the home position (to approximately 10 o’clock) directs the tip towards the septum (blue solid line) due to pre-shaped curvature. The blue dotted circle indicates perpendicular contact of the tip with the septal surface. (*C*) In dextrocardia, equivalent counterclockwise rotation (to approximately 8 o’clock) causes the shaft to contact the septum (red solid line), with the tip remaining parallel or deviated towards the free wall due to the fixed curvature (red dotted circle). (*D*) When the shaft contacts the septum, the delivery system should be slightly retracted to create a gap from the septal wall (red double-headed arrow), allowing further rotation without shaft impingement. (*E*) Additional counterclockwise rotation (to approximately 6 o’clock) then directs the tip towards the septum. The red dotted circle indicates perpendicular orientation of the tip to the septal surface. LA, left atrium; LV, left ventricle; RA, right atrium; RV, right ventricle.

Previous reports describing LP implantation in DC have noted challenges with catheter orientation.^4–6^ Our case builds on these observations by providing a detailed biomechanical explanation of how the pre-shaped curvature of the Micra delivery catheter necessitates additional counterclockwise torque to achieve septal deployment (*Figure 3*). In normal anatomy, the curvature facilitates septal orientation of the catheter tip: a small amount of clockwise torque typically directs the tip towards the septum after crossing the tricuspid valve (*Figure 3A* and *B*). In DC, however, an equivalent counterclockwise rotation causes the catheter shaft to contact the septum, while the pre-shaped tip may misalign or point towards free wall (*Figure 3C*). Slight retraction of the system creates a gap from the septal wall (*Figure 3D*), after which additional counterclockwise rotation with gentle advancement allows the tip—rather than the shaft—to engage the septum (*Figure 3E*). This manoeuvre goes beyond a simple mirror-image adjustment. Furthermore, operators should note that catheter shaft deflection may steer the tip away from the septum in DC, whereas it facilitates the septal alignment in normal anatomy.

The imaging strategy is also crucial. There are inconsistent interpretations among reports regarding the utility of horizontally flipped fluoroscopy in DC: one report described it as helpful for familiar projections,^5^ whereas another noted impaired tactile feedback and hand-eye coordination.^6^ Because catheter manipulation in DC requires more than a mirror-image adjustment, correct septal deployment may be more reliably achieved under standard non-flipped fluoroscopic orientation.

This case had several limitations. First, because this is a single case, the generalizability and clinical relevance of these technical insights are uncertain. In addition, anatomical configuration (dextroversion vs. DC + SI) may influence catheter dynamics, and therefore, the procedural considerations described here may not be fully applicable to all forms of DC. Second, the rationale for selecting an LP over a transvenous pacemaker was not explored. Long-term survival after a successful transvenous pacemaker implantation in DC is favourable,^7^ indicating that a transvenous device might have been a reasonable alternative. Finally, the choice between the Aveir (Abbott, Chicago, IL, USA) and the Micra was not discussed. Notably, the Aveir lacks a pre-shaped tip,^8,9^ which may offer advantages in navigating mirrored cardiac anatomy.

## Conclusions

We successfully performed Micra LP implantation in a patient with DC + SI. The three-dimensional curvature of the Micra delivery catheter posed challenges beyond simple mirror-image manipulation. Additional counterclockwise torque was essential to achieve correct septal positioning. Familiarity with the catheter’s design and torque behaviour was crucial for safe navigation and precise deployment in mirrored cardiac anatomy.

## Lead author biography


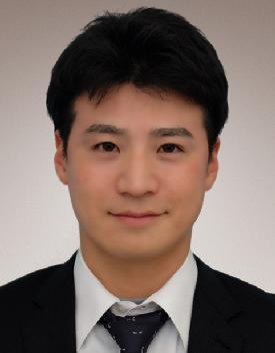


Dr Kohei Iwasa is a dedicated cardiologist specializing in arrhythmia, heart failure, and device therapy at the Department of Arrhythmia, Cardiovascular Center, Sakurabashi Watanabe Advanced Healthcare Hospital. He received his medical degree from Kobe University in 2017. From 2022 to 2025, he served as a cardiology staff member at the same institution. His current clinical and research interests focus on catheter ablation and device therapy.

## Data Availability

Data are not publicly available due to patient privacy but can be provided upon reasonable request.
